# Molecular and Genomic Mechanisms Linking Diabetes Mellitus and Periodontitis: From Pathogenesis to Translational Opportunities

**DOI:** 10.3390/ijms27177609

**Published:** 2026-08-25

**Authors:** Nozomi Harai, Kyoichiro Tsuchiya

**Affiliations:** 1 Department of Diabetes and Endocrinology, Graduate School of Interdisciplinary Research, Faculty of Medicine, University of Yamanashi, 1110 Shimokato, Chuo 409-3898, Yamanashi, Japan; 2 Department of Endocrinology and Metabolism, Toranomon Hospital, 2-2-2 Toranomon, Minato-ku, Tokyo 105-8470, Japan

**Keywords:** diabetes mellitus, periodontitis, inflammation, AGE–RAGE signaling, oral microbiome, genomics, epigenetics, single-cell RNA sequencing

## Abstract

Diabetes mellitus and periodontitis are bidirectionally associated chronic disorders linked through metabolic dysregulation, host inflammation, microbial dysbiosis, and impaired tissue remodeling. This review summarizes clinical, molecular, cellular, genomic, epigenomic, transcriptomic, and microbial evidence concerning the mechanisms underlying this relationship and their potential translational relevance. Chronic hyperglycemia is associated with advanced glycation end product signaling through the receptor for advanced glycation end products, mitogen-activated protein kinase/nuclear factor-κB activation, reactive oxygen species production, oxidative stress, and NLR family pyrin domain-containing 3 inflammasome activation, which may contribute to enhanced cytokine responses and periodontal tissue injury. Diabetes is also associated with altered neutrophil and macrophage function, increased T helper 17/interleukin-17 signaling, and an elevated receptor activator of nuclear factor-κB ligand/osteoprotegerin ratio, thereby favoring osteoclastogenesis and alveolar bone loss. Conversely, periodontal inflammation and microbial products may contribute to systemic low-grade inflammation, insulin resistance, and metabolic dysregulation. Multi-omics studies have identified shared susceptibility loci, regulatory networks, and disease-associated cell states, although their causal and clinical significance remains incompletely defined. These findings suggest potential roles for integrated medical–dental care, glycemic screening in dental settings, periodontal inflammation control, host-modulatory therapies, and regenerative biomaterials. Further longitudinal and experimental studies are needed to determine their clinical applicability.

## 1. Introduction

Periodontitis is a chronic inflammatory disease initiated by dysbiotic microbial communities; however, its progression is fundamentally shaped by reciprocal interactions between these communities and the host immune–inflammatory response. This “host–microbiome crosstalk” is critical for maintaining periodontal homeostasis, and its disruption—involving aberrant immune activation and dysbiosis—underlies the pathogenesis of tissue destruction [1]. In the current classification framework, diabetes mellitus (DM) is regarded not as a distinct periodontal disease entity, but as an important modifying factor that influences the progression and severity of periodontitis [1].

Epidemiological studies have demonstrated a bidirectional association between diabetes and periodontitis [2]. Notably, periodontitis exhibits a high comorbidity rate among diabetic patients, affecting a substantial proportion of this population. Diabetes, particularly when glycemic management is poor, is associated with increased periodontal inflammation, clinical attachment loss, alveolar bone loss, and tooth loss. Conversely, severe periodontitis is associated with an increased risk of incident diabetes and poorer glycemic management in individuals with established diabetes [3,4]. On the basis of these findings, international consensus recommendations emphasize the importance of coordinated medical and dental care [5]. However, the metabolic benefits of periodontal treatment vary among studies, and the factors underlying this heterogeneity remain incompletely understood.

At the biological level, this relationship involves complex interactions where chronic hyperglycemia and oxidative stress alter innate and adaptive immune responses. This local inflammatory environment subsequently disrupts bone metabolism, promotes oral microbiome dysbiosis, and ultimately contributes to systemic low-grade inflammation. Human studies provide evidence of clinical relevance, whereas animal models and in vitro studies complement our understanding of the underlying cellular and molecular mechanisms. More recently, genomic, transcriptomic, epigenomic, and microbiome analyses have begun to identify shared susceptibility factors, regulatory networks, and disease-associated cellular states.

This review summarizes the molecular, cellular, genomic, epigenomic, transcriptomic, and microbial mechanisms linking diabetes and periodontitis and discusses their relevance to integrated medical–dental care, glycemic screening, host-modulatory therapies, and regenerative approaches.

This review further distinguishes evidence derived from human studies, animal models, and in vitro experiments because these approaches answer different questions. Human studies define clinical associations and treatment responses, animal models test the contribution of specific pathways in an intact organism, and cell-culture systems isolate direct responses to glucose, advanced glycation end products (AGEs), cytokines, or microbial products. This evidence-based hierarchy is used to integrate established inflammatory mechanisms with emerging findings from single-cell analysis, epigenomics, microbiome research, and molecular comorbidity networks.

## 2. Literature Selection and Scope

To provide a comprehensive overview of the bidirectional relationship between diabetes mellitus and periodontitis, the relevant literature was surveyed using PubMed and Google Scholar. Focusing on core topics including hyperglycemia-driven pathways, immune dysregulation, and molecular mechanisms, this narrative review prioritizes landmark clinical studies, foundational experimental models, and authoritative consensus reports, selected based on our clinical and research expertise in the field.

## 3. Clinical Evidence for the Bidirectional Relationship

The clinical association between diabetes and periodontitis is relevant not only for risk assessment but also for evaluating whether controlling periodontal inflammation can influence systemic metabolic outcomes. Periodontal treatment has therefore been examined as a potential component of comprehensive diabetes care.

A 2022 Cochrane review reported that, compared with no treatment or usual care, periodontal treatment reduced glycated hemoglobin (HbA1c) by an average of 0.43 percentage points at 3–4 months and 0.30 percentage points at 6 months in people with diabetes [2]. In a 12-month single-center randomized trial, HbA1c was 0.6 percentage points lower in the intensive periodontal treatment group than in the control group [6]. In contrast, another large multicenter trial showed that, although periodontal status improved, the between-group difference in the change in HbA1c at 6 months was −0.05 percentage points (95% confidence interval, −0.23 to 0.12), indicating no clear improvement in glycemic management [7]. This clinical heterogeneity stems primarily from variations in baseline periodontal inflammation, metabolic status, treatment intensity, follow-up duration, and concurrent changes in diabetes therapy. Therefore, the value of periodontal assessment and treatment in diabetes care should be considered in terms of broader health benefits, including control of oral inflammation and preservation of teeth, rather than solely in terms of HbA1c reduction.

Observational studies consistently relate diabetes, particularly inadequate glycemic management, to greater periodontal severity and progression, but obesity, smoking, socioeconomic conditions, oral hygiene, medication use, and healthcare access remain potential confounders. Periodontal intervention trials provide stronger evidence for a treatment effect, although the magnitude and persistence of HbA1c improvement vary with baseline periodontal inflammation, metabolic status, treatment intensity, follow-up duration, and concomitant changes in diabetes therapy [2,6,7,8,9,10]. Accordingly, the clinical data establish a reproducible association and a modest short-term treatment effect at the population level, whereas experimental studies are required to identify the pathways responsible for these observations.

## 4. Hyperglycemia-Driven Inflammatory Signaling

### 4.1. AGE–RAGE/MAPK/NF-κB Signaling and Oxidative Stress

The AGE–receptor for advanced glycation end products (RAGE) signaling axis is a central molecular connection between chronic hyperglycemia and periodontal inflammation. AGEs accumulate during sustained hyperglycemia and interact with RAGE, promoting redox-sensitive and inflammatory signaling. Consistent with these clinical findings, in human gingival samples, *AGER* messenger RNA (mRNA) expression was significantly higher in participants with type 2 diabetes mellitus (T2DM) and periodontitis than in nondiabetic participants with periodontitis [11]. Additionally, recent clinical studies have shown that the oral microbiome of type 1 diabetes mellitus (T1DM) patients undergoes shifts in diversity and abundance, with elevated levels of specific pathogens such as *Porphyromonas gingivalis* and *Prevotella intermedia* [12].

At the cellular level, AGE exposure increases interleukin-6 (IL-6) and intercellular adhesion molecule 1 (ICAM-1) expression in human gingival fibroblasts through RAGE, mitogen-activated protein kinase (MAPK) and nuclear factor-κB (NF-κB). AGE exposure also amplifies intracellular reactive oxygen species (ROS) generation and promotes monocytic cell adhesion [13]. Although these in vitro findings indicate that hyperglycemia-derived molecular products can directly alter the inflammatory phenotype of resident periodontal cells, their relative contribution to the complex disease environment in vivo remains partially speculative and requires further clinical validation.

### 4.2. NLRP3 Activation and Cytokine Amplification

Hyperglycemia also enhances inflammasome-related signaling in periodontal tissues. High-glucose conditions have been shown to exacerbate NLR family pyrin domain-containing 3 (NLRP3)-related inflammatory responses in gingival epithelial cells [14]. In human gingival tissues, NLRP3 and interleukin-1β (IL-1β) were upregulated in the gingival epithelium of individuals with periodontitis and T2DM [14,15].

Together, AGE–RAGE signaling, MAPK/NF-κB activation, ROS production, oxidative stress, and NLRP3 activation provide a mechanistic basis for the cytokine-rich periodontal microenvironment observed in diabetes. These pathways promote the production of IL-1β, IL-6, tumor necrosis factor-α (TNF-α), and related inflammatory mediators, thereby contributing to immune-cell recruitment, tissue destruction, and impaired periodontal repair [16,17].

### 4.3. Experimental Support from Animal and In Vitro Models

Mechanisms demonstrated in preclinical animal models include the investigation of AGE–RAGE signaling and interleukin-17 (IL-17)-mediated immune–microbial interactions in diabetes-associated periodontitis. In preclinical research using streptozotocin-induced diabetic mice infected with *Porphyromonas gingivalis*, administration of soluble RAGE (sRAGE) reduced alveolar bone loss in a dose-dependent manner. Gingival tissues from sRAGE-treated mice also showed decreased production of TNF-α and IL-6, lower matrix metalloproteinase levels, and reduced AGE accumulation [18]. In contrast, sRAGE administration did not reduce alveolar bone loss in normoglycemic mice. These findings demonstrated the involvement of RAGE-mediated inflammatory responses in alveolar bone loss in diabetic mice. While these studies suggest a causal link between diabetes-induced immune dysregulation and bone loss, further clinical investigation is needed to determine the exact extent to which these specific pathways operate in human patients.

In another diabetic mouse model, increased IL-17 expression in periodontal tissues, enhanced neutrophil recruitment, and changes in oral microbial composition were observed. Transfer of the oral microbiota from diabetic mice to normoglycemic germ-free recipients increased periodontal neutrophil recruitment, IL-6 and receptor activator of nuclear factor-κB ligand (RANKL) expression, and alveolar bone resorption compared with transfer of microbiota from normoglycemic mice. Treatment of diabetic donor mice with an anti-IL-17 antibody attenuated the changes in oral microbial composition. Transfer of the microbiota obtained after anti-IL-17 treatment also resulted in reduced neutrophil recruitment, IL-6 and RANKL expression, and alveolar bone resorption in germ-free recipients [19].

In vitro studies have examined the effects of high glucose and AGEs on periodontal tissue-resident cells. In human gingival fibroblasts, AGE stimulation increased the mRNA and protein expression of RAGE, IL-6, and ICAM-1, increased intracellular ROS production, and promoted the adhesion of THP-1 human monocytic cells to gingival fibroblasts. Small interfering RNAs targeting RAGE or IL-6 and inhibitors of p38 MAPK, extracellular signal-regulated kinase (ERK) MAPK, and NF-κB suppressed AGE-induced IL-6 and ICAM-1 expression. IL-6 small interfering RNA also reduced AGE-induced THP-1 cell adhesion [13]. In human gingival epithelial cells, high-glucose conditions enhanced NLRP3-related inflammatory responses [14]. These experiments demonstrated that AGEs and high glucose, two features of the diabetic environment, alter inflammatory signaling and cellular adhesion responses in periodontal tissue-resident cells.

In summary, evidence from human tissues, animal models, and in vitro studies suggests that hyperglycemia-driven pathways—including AGE–RAGE signaling, oxidative stress, and inflammasome activation—may contribute to the pro-inflammatory microenvironment and impaired tissue repair observed in diabetic periodontal disease, although further clinical validation is required to establish their definitive impact.

## 5. Host Immune Dysregulation and Bone Remodeling Imbalance

### 5.1. Immune-Cell Dysfunction and IL-17 Signaling

Diabetes changes both the protective and tissue-damaging functions of neutrophils in periodontal tissues. Reported abnormalities include impaired chemotaxis, phagocytosis, and intracellular microbial killing, which reduce the efficiency of microbial clearance. At the same time, increased production of reactive oxygen species, proteolytic enzymes, and neutrophil extracellular traps can damage surrounding periodontal cells and extracellular matrix. Thus, reduced antimicrobial control and increased inflammatory injury can occur together in the diabetic periodontal environment [20].

T helper 17 (Th17) cells connect immune activation with neutrophil recruitment and bone remodeling through interleukin-17 (IL-17). IL-17 stimulates epithelial and stromal cells to produce chemokines that recruit neutrophils to periodontal tissues. It also increases inflammatory mediator and RANKL expression, thereby linking persistent immune activation with osteoclast formation. In diabetes, increased IL-17 signaling has been reported together with periodontal inflammation, altered host–microbial interactions, and increased alveolar bone loss [9,19].

Macrophages participate in both the initiation and resolution of periodontal inflammation. As detailed in Section 4, their activation by hyperglycemia, AGEs, and microbial ligands amplifies the production of pro-inflammatory cytokines and RANKL, further promoting osteoclastogenic signaling. Diabetes also alters macrophage functions involved in tissue homeostasis, such as debris removal, angiogenesis, and matrix remodeling. Consequently, the disruption of these processes prolongs inflammation while limiting tissue repair [21].

### 5.2. RANKL/OPG Imbalance and Osteoclastogenesis

Periodontal tissue destruction ultimately reflects an imbalance between inflammatory injury and tissue repair. In T1DM- and T2DM-associated periodontitis, sustained inflammation and immune-cell activation are accompanied by an increased RANKL/osteoprotegerin (OPG) ratio, which favors osteoclast differentiation and activation. Enhanced osteoclastogenesis promotes alveolar bone resorption and contributes to clinical attachment loss and tooth-supporting tissue destruction [12,16,17].

Diabetes also suppresses the tissue-repair arm of periodontal homeostasis. Hyperglycemia and oxidative stress reduce proliferation, migration, and differentiation of periodontal ligament cells, gingival fibroblasts, endothelial cells, and osteoblast-lineage cells; increased apoptosis further limits regenerative capacity [16,20]. Consequently, alveolar bone loss reflects both enhanced osteoclastogenesis and inadequate osteoblast-mediated bone formation. Vascular dysfunction and reduced angiogenic responses restrict oxygen and nutrient delivery during healing. Thus, the diabetic periodontal phenotype is characterized by both accelerated inflammatory tissue destruction and impaired resolution of inflammation and regeneration. Therefore, in addition to controlling inflammation, restoring vascular function and tissue repair capacity represents an important therapeutic consideration. Although T1DM and T2DM have distinct upstream pathophysiological backgrounds, with autoimmune β-cell destruction and insulin deficiency characterizing T1DM and insulin resistance characterizing T2DM, available evidence suggests that several downstream mechanisms relevant to periodontal disease, including oxidative stress, immune dysregulation, and altered bone remodeling, may overlap between the two diabetes subtypes [12,20]. However, the relationship between periodontitis and T2DM has been more extensively studied than that with T1DM [12].

**Figure 1:**
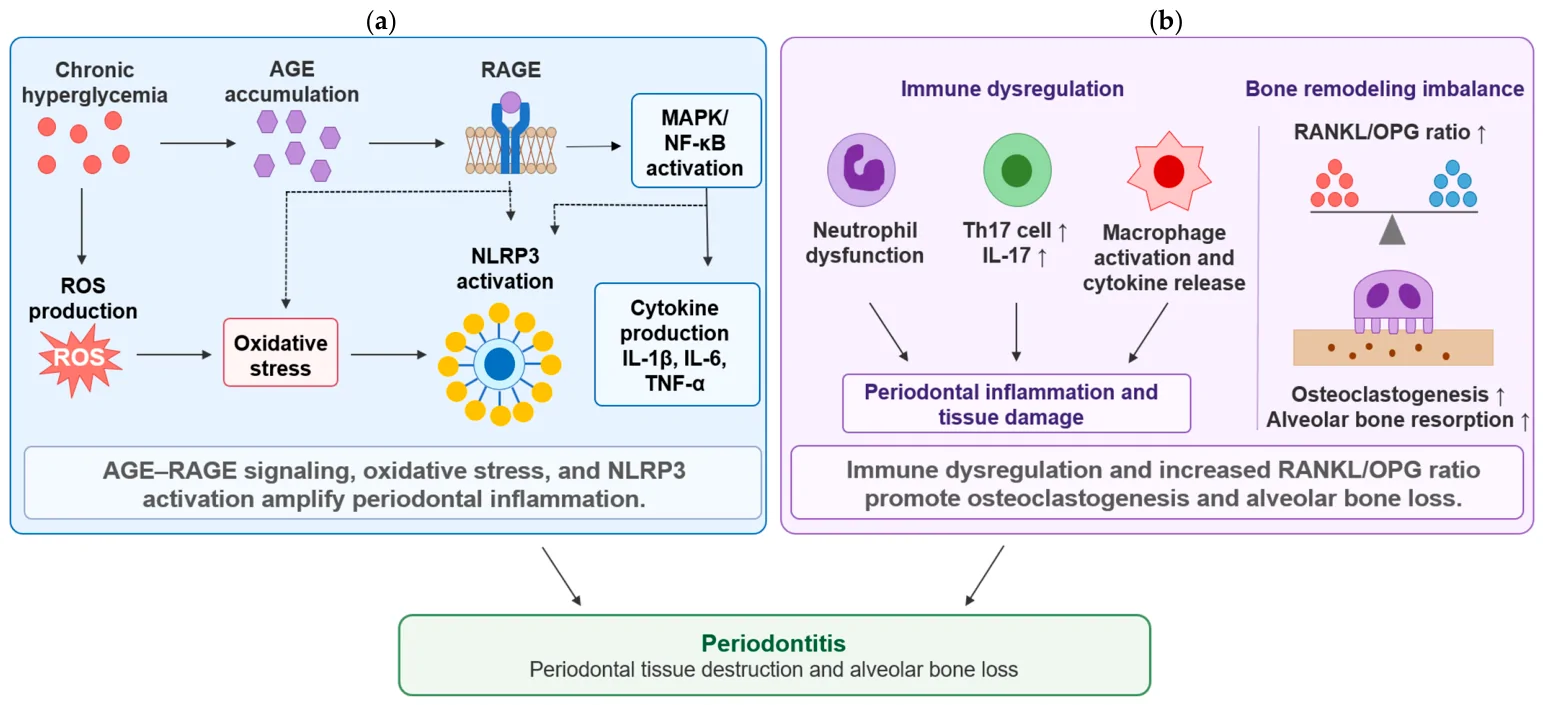
Hyperglycemia-driven periodontal inflammation and bone loss. (**a**) Chronic hyperglycemia promotes advanced glycation end product accumulation and receptor for advanced glycation end product signaling, leading to mitogen-activated protein kinase/nuclear factor-κB activation, reactive oxygen species production, oxidative stress, NLR family pyrin domain-containing 3 inflammasome activation, and the production of inflammatory cytokines, including interleukin-1β, interleukin-6, and tumor necrosis factor-α. (**b**) Diabetes-associated immune dysregulation includes neutrophil dysfunction, enhanced T helper 17/interleukin-17 signaling, and macrophage activation. These alterations, together with an increased receptor activator of nuclear factor-κB ligand/osteoprotegerin ratio, favor osteoclastogenesis, alveolar bone resorption, and periodontal tissue destruction. Upward arrows indicate an increase. Abbreviations: AGE, advanced glycation end product; IL, interleukin; MAPK, mitogen-activated protein kinase; NF-κB, nuclear factor-κB; NLRP3, NLR family pyrin domain-containing 3; OPG, osteoprotegerin; RAGE, receptor for advanced glycation end products; RANKL, receptor activator of nuclear factor-κB ligand; ROS, reactive oxygen species; Th17, T helper 17; TNF-α, tumor necrosis factor-α.

Collectively, hyperglycemia-driven inflammatory signaling and diabetes-associated immune and bone-remodeling abnormalities form an interconnected pathway linking diabetes to periodontal inflammation and alveolar bone loss. Figure 1a summarizes AGE–RAGE signaling, oxidative stress, NLRP3 inflammasome activation, and cytokine production, whereas Figure 1b illustrates immune-cell dysfunction, Th17/IL-17 signaling, RANKL/OPG imbalance, osteoclastogenesis, and alveolar bone resorption.

### 5.3. PPAR-γ and Metabolic Nuclear Receptor Signaling

Metabolic nuclear receptors provide another interface between systemic metabolism and periodontal inflammation. Peroxisome proliferator-activated receptor gamma (PPAR-γ) is a transcription factor involved in adipogenesis, insulin sensitivity, lipid metabolism, and immune and inflammatory responses. Periodontitis and T2DM are both associated with activation of major inflammatory signaling pathways, including NF-κB, activator protein-1, and signal transducer and activator of transcription (STAT) proteins, thereby contributing to a reinforcing cycle of inflammation and metabolic dysfunction [22].

PPAR-γ activation can inhibit these pathways and suppress downstream inflammatory mediators such as TNF-α and IL-6 [22]. Although conventional PPAR-γ agonists such as thiazolidinediones are limited by adverse effects, selective PPAR-γ modulators may represent a host-modulatory strategy for future periodontal and metabolic disease management. However, these approaches remain largely at the exploratory preclinical stage with respect to periodontal outcomes, and further studies are required before their clinical relevance can be established.

## 6. Oral Microbiome Dysbiosis and Host–Microbe Interactions

The progression of periodontitis is largely shaped by reciprocal interactions between dysbiotic microbial communities and host immune/inflammatory factors. This dynamic host–microbiome crosstalk can be modulated by various host-derived factors, such as signaling molecules, which may differentially modulate oral microbiota and inflammatory responses depending on the stage of disease progression [23].

Diabetes may disrupt the balance of the oral microbiome by altering the local nutritional environment, inflammatory status, epithelial barrier function, immune responses, and tissue oxygenation in periodontal tissues. Clinical studies using metagenomic and 16S ribosomal RNA (rRNA) sequencing have reported changes in the composition of the subgingival microbiota in individuals with T2DM [24,25,26]. These alterations, often described as dysbiosis, are characterized by an increased abundance of pathogenic species and a reduction in beneficial commensals, which exacerbates periodontal destruction [27]. Furthermore, the interplay between the oral microbiome and the host is critical; oral dysbiosis facilitates systemic translocation of bacteria and their products, thereby intensifying systemic inflammation and worsening glycemic control [27,28]. In addition, microbial products such as lipopolysaccharide (LPS) and fimbriae can stimulate immune and epithelial cells through pattern-recognition receptors, including Toll-like receptors (TLRs), thereby further amplifying inflammatory responses.

**Figure 2:**
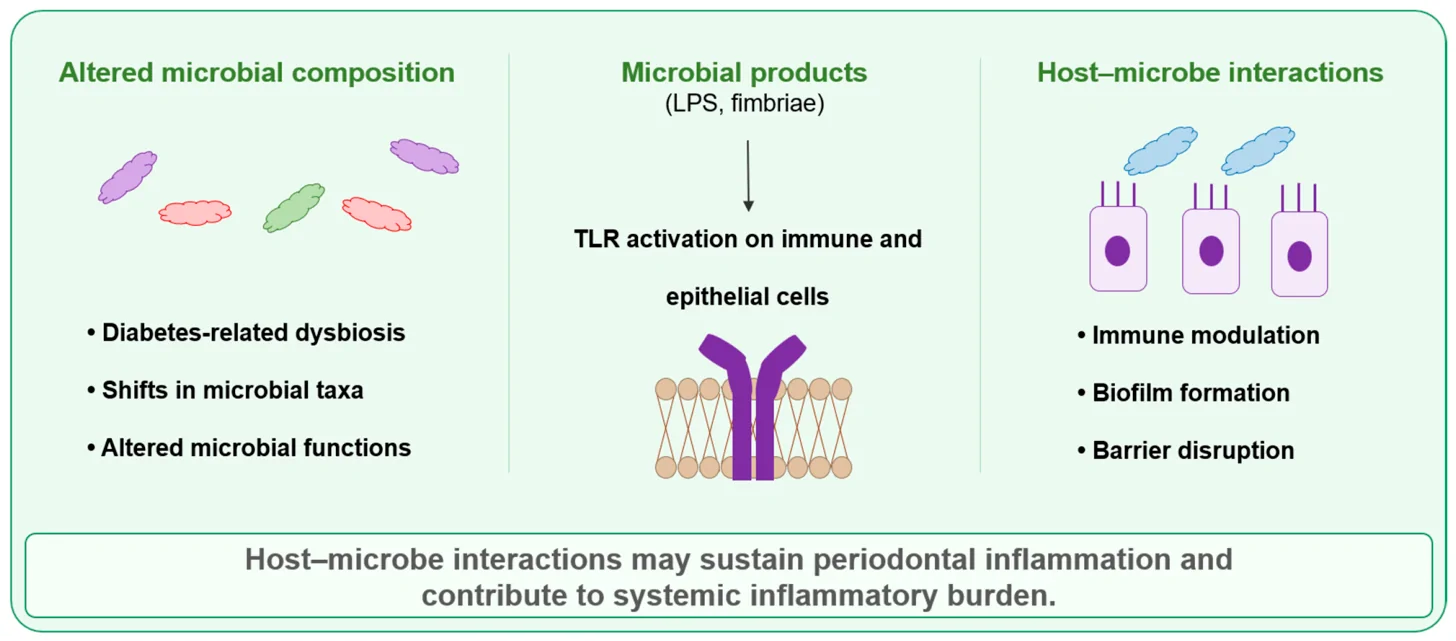
Oral dysbiosis and host–microbe interactions. Diabetes-associated changes in the periodontal microenvironment are linked to alterations in microbial composition and function. Microbial products, including lipopolysaccharide and fimbriae, activate Toll-like receptors on immune and epithelial cells and influence immune responses, biofilm formation, and epithelial barrier integrity. These host–microbe interactions may sustain periodontal inflammation and contribute to systemic inflammatory burden. Abbreviations: LPS, lipopolysaccharide; TLR, Toll-like receptor.

However, periodontitis in patients with diabetes cannot be explained simply by an increase in conventionally recognized periodontal pathogens. Indeed, individuals with T2DM and severe periodontitis have been reported to harbor lower levels or proportions of some traditional periodontal pathogens than normoglycemic individuals with comparable periodontitis severity [29]. This finding suggests that diabetes-associated immune and metabolic abnormalities may increase the inflammatory susceptibility of periodontal tissues independently of an increased bacterial burden. Therefore, the pathogenicity of the oral microbiome in diabetes-associated periodontitis is better understood in the context of host–microbiome interactions involving the metabolic environment, epithelial barrier, and host immunity, rather than solely by the presence or absence of specific bacterial species. These alterations in microbial composition and function, together with microbial products, TLR signaling, and disruption of host barrier and immune responses, are summarized in Figure 2.

Microbiome results also depend on the biological layer being measured. Amplicon-based taxonomic studies describe community composition, whereas shotgun metagenomics identifies microbial genes and functional potential. Metatranscriptomic approaches additionally measure which microbial genes are actively transcribed. Communities with similar taxonomic profiles can therefore differ in virulence, metabolism, and host-interacting functions. Conversely, current studies demonstrate diabetes-associated changes in periodontal microbial ecology but do not define a universal microbial signature of diabetes-associated periodontitis. These differences and apparent taxonomic variation can be significantly influenced by periodontal severity, glycemic status, medication use, diet, smoking, oral hygiene, sampling depth, sequencing platform, and analytical approaches [8,9,25,26,29,30]. Future studies that longitudinally assess microbial gene expression, the local nutrient environment, epithelial barrier function, host immunity, and clinical parameters, in addition to microbial composition, may help clarify the temporal relationship between dysbiosis and inflammation.

## 7. Genomic, Epigenomic, and Transcriptomic Regulation

Genomic, epigenomic, and transcriptomic approaches describe distinct layers of the diabetes–periodontitis relationship, ranging from inherited susceptibility to dynamic gene-expression patterns and cell-specific responses. These multi-omics analyses, when integrated with clinical phenotypes, allow us to distinguish shared systemic inflammatory signals from those occurring specifically within periodontal tissues.

### 7.1. Host Genetic Architecture and Shared Susceptibility

Susceptibility to diabetes and periodontitis is influenced by complex polygenic backgrounds and gene–environment interactions. Candidate-gene studies have investigated inflammatory and immune-related genes, including *IL1B*, *TNF*, *IL6*, and *AGER* [20,31]. Human genome-wide association studies (GWAS) support a complex polygenic contribution to periodontitis susceptibility [32].

Cross-trait genomic analyses have identified shared genetic architecture between periodontal disease, T2DM, and related glycemic traits. Wu et al. identified 62 independent pleiotropic loci shared between periodontal disease and T2DM or related glycemic traits and reported that some of these genetic associations were independent of body mass index [33]. Mendelian randomization analyses have also suggested a causal role for genetically elevated fasting glucose in increasing periodontitis susceptibility [34]. These findings strengthen the biological plausibility of a link between glycemic dysregulation and periodontal disease risk.

Network analyses extend the concept of shared susceptibility from individual loci to molecular pathways. A periodontitis-centered molecular comorbidity network identified shared genes and protein–protein interactions linking periodontitis with metabolic, cardiovascular, immune, and inflammatory diseases [31]. Molecules associated with diabetes and periodontitis overlapped in pathways related to inflammatory signaling, oxidative stress, immune regulation, vascular function, and tissue remodeling. Although such networks are useful for organizing candidate pathways, clarifying their biological relevance requires integration with gene expression in periodontal tissues, single-cell profiles, epigenomic data, and longitudinal clinical phenotypes.

### 7.2. Bulk and Single-Cell Transcriptomics

Transcriptomic approaches have expanded research on diabetes-associated periodontitis from the analysis of individual molecular pathways to network-level analyses involving multiple pathways and cell populations. On the other hand, findings from single-cell RNA sequencing (scRNA-seq) of human gingival biopsy specimens represent an emerging area of research; for instance, Agrafioti et al. identified multiple macrophage populations in clinically healthy and periodontitis-affected gingival tissues [35]. Transcriptional regulator activity in periodontal macrophages also differed according to the presence or absence of T2DM. These macrophages exhibited diverse transcriptional states that were not adequately represented by a simple division into classically activated M1 and alternatively activated M2 macrophages.

In addition, bulk transcriptomic analyses using public datasets have identified pathways shared by periodontitis and T2DM, including leukocyte migration, chemokine signaling, and immune activation [36]. These analyses are useful for identifying candidate molecular networks. Whether these transcriptional changes reflect causal mechanisms, compensatory responses, or treatment responses may be clarified by combining experimental validation with longitudinal human studies.

### 7.3. Non-Coding RNA and Epigenetic Regulation

Non-coding RNAs and epigenetic regulation are important mechanisms linking diabetes-associated metabolic abnormalities to inflammatory gene expression in periodontal tissues. Gingival crevicular fluid levels of microRNA-146a (miR-146a) and microRNA-155 (miR-155) have been reported to increase in periodontitis and decrease after nonsurgical periodontal treatment, regardless of the presence or absence of T2DM [37]. These findings suggest that these microRNAs may reflect inflammatory activity and treatment response in periodontal tissues.

Epigenetic changes, such as DNA methylation, chromatin remodeling, and histone modification, may contribute to sustained pro-inflammatory gene-expression patterns in periodontal tissues under diabetic conditions [38,39]. Future integration of epigenomic profiling with scRNA-seq, spatial transcriptomics, proteomics, and metagenomics may further clarify cell-specific regulatory mechanisms in diabetes-associated periodontal disease.

## 8. Periodontitis-Driven Systemic Inflammation and Metabolic Dysregulation

The bidirectional relationship between diabetes and periodontitis necessitates an understanding of how periodontal inflammation influences systemic metabolic regulation. The ulcerated periodontal pocket epithelium serves as a critical interface through which locally produced inflammatory mediators—specifically IL-1β, IL-6, and TNF-α—along with microbial products, can access the systemic circulation without requiring continuous bacteremia [5,30].

Once in the circulation, these factors disrupt metabolic homeostasis through several experimentally supported pathways. In the liver, skeletal muscle, and adipose tissue, this inflammatory signaling interferes with insulin-receptor pathways, thereby promoting insulin resistance [8,9,21]. Specifically, hepatic responses are characterized by increased acute-phase protein production, while adipose-tissue inflammation alters adipokine release, cumulatively increasing the systemic inflammatory burden already present in T2DM [8,9,21].

Furthermore, microbial products, including circulating LPS and other pathogen-associated molecular patterns, directly activate TLR signaling in immune and metabolic tissues, reinforcing innate immune activation [8,9,21]. While the relative clinical contribution of these pathways in humans remains incompletely quantified, the convergence of inflammatory mediators and reprogrammed innate immunity provides a coherent biological framework linking localized periodontal inflammation to systemic metabolic dysregulation [8,9,21].

## 9. Translational and Clinical Implications

### 9.1. Integrated Periodontal and Diabetes Care

Medical–dental collaboration is one practical approach to translating knowledge of the association between diabetes and periodontitis into clinical practice. Incorporating assessment of periodontal status and, when appropriate, recommending dental consultation into diabetes care, while recognizing potential metabolic risk based on oral findings and medical history in dental practice, may facilitate timely referrals between medical and dental professionals. Diabetes care providers can also support oral health by asking patients about gingival bleeding, tooth mobility, oral discomfort, and regular dental attendance. International consensus recommendations support this collaborative approach [5].

To facilitate interprofessional collaboration in routine clinical practice, simple referral pathways, shared educational materials, and consistent communication emphasizing that periodontal health is an integral part of comprehensive diabetes management may be helpful. Such implementation strategies may be particularly useful in community healthcare settings where patients regularly visit dental clinics but do not undergo routine metabolic screening.

### 9.2. Glycemic Screening and Risk Assessment in Dental Settings

Dental clinics provide an opportunity to identify individuals who may not receive regular medical screening. In our multicenter prospective Kyoutou Dental and Diabetes (KDD) Study, 749 dental patients who were not receiving diabetes treatment underwent capillary blood glucose screening. Of these participants, 139 (18.6%) had a positive screening result. Among the 34 participants who subsequently attended an internal medicine clinic, eight were diagnosed with diabetes and 10 with prediabetes. Bleeding on probing and radiographic alveolar bone loss were independently associated with positive screening results [40].

These findings support the potential use of dental settings for the early detection of dysglycemia. Future implementation studies evaluating screening thresholds, referral pathways, patient acceptability, cost-effectiveness, and integration with local healthcare networks are expected to help identify feasible models of care.

### 9.3. Host-Modulatory Metabolic Therapies

Metabolic therapies may influence periodontal tissues both indirectly, through improved glycemic management and weight reduction, and directly, through effects on inflammation, oxidative stress, immune regulation, and bone metabolism. Glucagon-like peptide-1 receptor agonists (GLP-1RAs) improve glycemic management and promote weight loss. Preclinical studies and limited emerging clinical evidence suggest that GLP-1RAs may influence periodontal inflammation, immune regulation, and bone metabolism; however, robust evidence from clinical trials using standardized periodontal outcomes remains limited [41]. Therefore, the clinical efficacy of GLP-1RAs for periodontal outcomes remains to be established in appropriately designed clinical trials.

PPAR-γ-directed approaches also represent a potential host-modulatory strategy, given the role of PPAR-γ in metabolic and inflammatory gene regulation [22]. At present, these approaches remain entirely at the exploratory preclinical stage, and their clinical significance for periodontal outcomes has not been established. Incorporating standardized periodontal assessments into future trials of metabolic therapies would facilitate evaluation of their effects on periodontal disease activity and treatment response.

### 9.4. Regenerative Biomaterials and Tissue Engineering

Advanced biomaterials offer innovative approaches to periodontal regeneration within the repair-compromising diabetic microenvironment. Hyperglycemia, oxidative stress, chronic inflammation, impaired angiogenesis, and altered immune-cell function can limit tissue repair. Microenvironment-responsive biomaterials are therefore being developed to modulate inflammatory and oxidative stress pathways while supporting tissue regeneration.

For example, a calcitonin gene-related peptide-loaded, ROS-responsive hydrogel restored neuro-angiogenic signaling and promoted bone regeneration in a diabetic mouse model of periodontitis [42]. Release kinetics, biocompatibility, degradation behavior, antimicrobial properties, and compatibility with periodontal surgical procedures are important considerations for future clinical translation.

## 10. Future Perspectives

**Figure 3:**
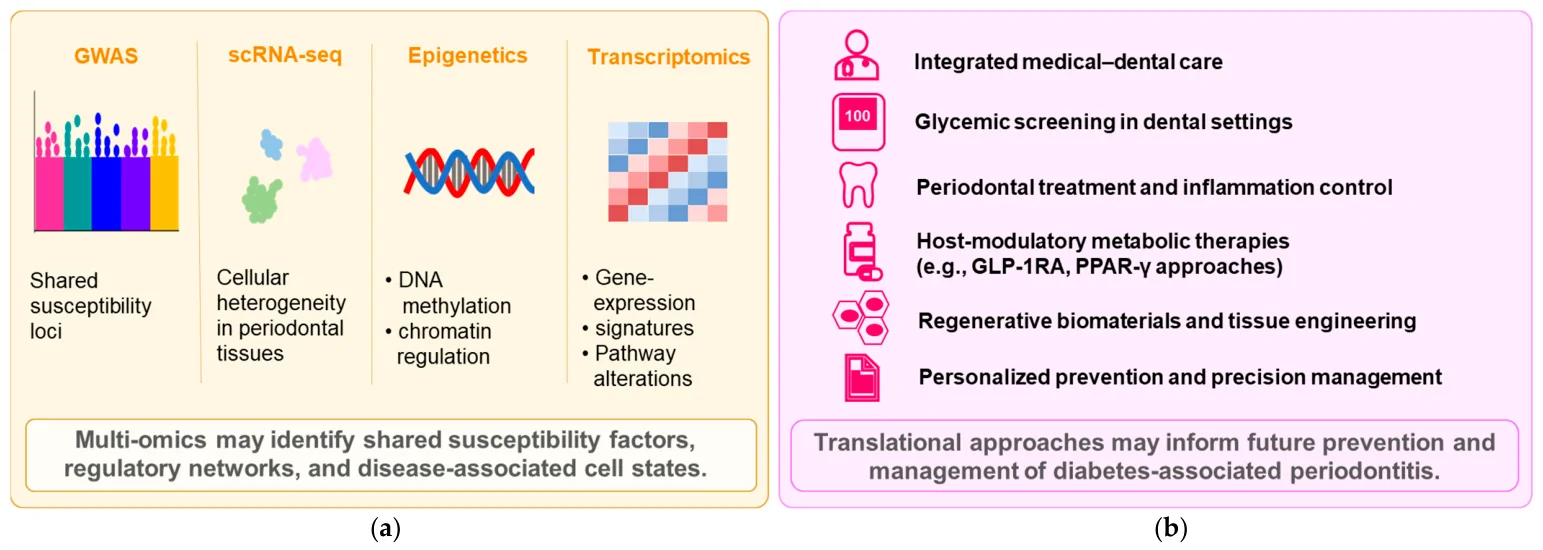
Multi-omics and translational applications. (**a**) Genomic, epigenomic, transcriptomic, and single-cell approaches provide information on shared susceptibility loci, cellular heterogeneity, gene-expression patterns, and regulatory mechanisms in diabetes-associated periodontitis. Integration of these approaches with clinical phenotyping and experimental validation may help identify candidate biomarkers, regulatory networks, and disease-associated cell states. (**b**) Findings derived from multi-omics analyses may inform integrated medical–dental care, glycemic screening and risk assessment in dental settings, periodontal inflammation control, evaluation of host-modulatory metabolic therapies, and the development of regenerative biomaterials and tissue-engineering approaches. Abbreviations: GLP-1RA, glucagon-like peptide-1 receptor agonist; GWAS, genome-wide association study; PPAR-γ, peroxisome proliferator-activated receptor gamma; scRNA-seq, single-cell RNA sequencing.

Future research may benefit from linking clinical phenotyping, multi-omics analyses, and experimental validation within a coordinated research framework. Longitudinal human studies incorporating standardized periodontal and metabolic assessments and serial sample collection may help identify reproducible biomarkers, characterize patient subgroups with different treatment responses, and examine the temporal relationship between molecular changes and disease progression. Figure 3a presents molecular findings derived from genomic, epigenomic, transcriptomic, and single-cell analyses, whereas Figure 3b outlines the potential clinical and regenerative applications associated with these findings.

Combining single-cell RNA sequencing (scRNA-seq), spatial transcriptomics, epigenomics, proteomics, metabolomics, and microbiome analyses may provide insights into the relationship between systemic metabolic status and cellular responses in periodontal tissues at the molecular, cellular, and tissue levels. Clinical cohorts may first help identify patients more likely to develop severe periodontitis and subgroups with different treatment responses. Multi-omics analyses could then be used to explore the molecular pathways and cellular states associated with these clinical patterns. Single-cell and spatial analyses may help identify the cell types and tissue locations in which candidate alterations occur. Cell culture systems, organotypic models, and animal models may provide complementary approaches for examining the functional effects of candidate pathways on inflammation, host–microbial interactions, and bone remodeling.

The interpretation of molecular findings may also be influenced by diabetes type and duration, long-term glycemic exposure, short-term glycemic variability, obesity, smoking, medication use, periodontitis stage and grade, and periodontal sampling site. Consistent documentation of these factors, together with evaluation in independent human populations using standardized measurement methods, may help distinguish changes associated with diabetes-related periodontitis from those common to inflammation more broadly. Such studies may also clarify the potential clinical relevance of candidate biomarkers and therapeutic targets.

From a translational perspective, future areas of investigation may include the effects of metabolic therapies on periodontal outcomes, glycemic screening and referral pathways in dental settings, and regenerative approaches that account for the inflammatory and oxidative-stress environment associated with diabetes. Further clinical and experimental evidence in these areas may support the development of preventive strategies and more individualized management of diabetes-associated periodontitis.

## 11. Conclusions

DM and periodontitis are interconnected through multiple pathways involving metabolism, inflammation, microbes, genomics, and tissue remodeling. Hyperglycemia promotes AGE–RAGE signaling, MAPK/NF-κB activation, ROS production, oxidative stress, NLRP3 activation, and cytokine production. These changes may affect immune cell function, Th17/IL-17 signaling, macrophage activation, and the RANKL/OPG balance, thereby contributing to osteoclastogenesis and alveolar bone resorption.

Oral microbiome dysbiosis, changes in microbial composition and function, microbial products, and TLR-mediated host–microbe interactions may also contribute to the persistence of periodontal inflammation. In addition, genomic, epigenomic, transcriptomic, single-cell, and metagenomic analyses are gradually revealing shared susceptibility factors, regulatory networks, and disease-associated cellular states underlying the relationship between DM and periodontitis.

These findings may help support medical–dental collaboration, glycemic screening and risk assessment in dental practice, periodontal treatment, inflammation control, therapies that consider host responses, and the development of regenerative biomaterials. Continued integration of clinical data, multi-omics analyses, and experimental validation is expected to facilitate the translation of these mechanistic findings into preventive and individualized management of diabetes-associated periodontal disease.

## Data Availability

No new data were created or analyzed in this study. Data sharing is not applicable to this article.

## Abbreviations

The following abbreviations are used in this manuscript:

AGE	Advanced glycation end product
DM	Diabetes mellitus
ERK	Extracellular signal-regulated kinase
GLP-1RA	Glucagon-like peptide-1 receptor agonist
GWAS	Genome-wide association study
HbA1c	Glycated hemoglobin
ICAM-1	Intercellular adhesion molecule 1
IL	Interleukin
LPS	Lipopolysaccharide
MAPK	Mitogen-activated protein kinase
mRNA	Messenger RNA
NF-κB	Nuclear factor-κB
NLRP3	NLR family pyrin domain-containing 3
OPG	Osteoprotegerin
PPAR-γ	Peroxisome proliferator-activated receptor gamma
RAGE	Receptor for advanced glycation end products
RANKL	Receptor activator of nuclear factor-κB ligand
ROS	Reactive oxygen species
rRNA	Ribosomal RNA
scRNA-seq	Single-cell RNA sequencing
STAT	Signal transducer and activator of transcription
T1DM	Type 1 diabetes mellitus
T2DM	Type 2 diabetes mellitus
Th17	T helper 17
TLR	Toll-like receptor
TNF-α	Tumor necrosis factor-α
